# Do adolescents exposed to peer aggression at school consider themselves to be victims of bullying? The influence of sex and age

**DOI:** 10.47626/2237-6089-2021-0219

**Published:** 2022-05-26

**Authors:** Marlene A. Vieira, Bjørn H. Handegård, John A. Rønning, Cristine S. Duarte, Jair J. Mari, Isabel A. Bordin

**Affiliations:** 1 Universidade Federal de São Paulo São Paulo SP Brazil Universidade Federal de São Paulo , São Paulo , SP , Brazil .; 2 University of Tromsø Tromsø Norway University of Tromsø , Tromsø , Norway .; 3 New York State Psychiatric Institute Columbia University New York NY USA New York State Psychiatric Institute , Columbia University , New York , NY , USA .

**Keywords:** Adolescent, bullying, aggression, sex, age groups

## Abstract

**Introduction:**

Exposure to peer aggression (PA) and bullying victimization (BV) are both expressions of peer victimization.

**Objectives:**

In four age-sex groups, (1) Can exposure to PA and BV be considered distinct experiences? (2) To what extent do adolescents exposed to PA consider themselves bullying victims? and (3) What is the effect on BV of the number of PA events experienced?

**Methods:**

This cross-sectional study evaluated a probabilistic community-based sample of 669 adolescents (11-15 years, 51.7% girls). A three-stage probabilistic sampling plan involved random selection of census units, eligible households, and one target child per household selected. A 15-item scale investigated exposure to PA events (physical aggression, verbal harassment, social manipulation) occurring more than once in the past six months. BV occurring more than once a week or most days in the past six months was investigated after presenting respondents with a BV definition that required them to feel harmed by their victimization experiences.

**Results:**

Adolescents exposed to PA and/or BV reported PA only (76.2%), BV only (4.7%), and both (19.1%). Rates of BV among those exposed to PA were as follows: 11-to-12-year-old boys (22.7%), 13-to-15-year-old boys (9.7%), 11-to-12-year-old girls (46.5%), and 13-to-15-year-old girls (13.2%). Multiple logistic regression analysis (outcome = BV) found a significant interaction between PA, age, and sex. PA events had a significant effect on BV for all except older girls.

**Conclusion:**

Exposure to PA and BV are different constructs; few older boys exposed to PA consider themselves bullying victims; and older girls are less affected by PA when it comes to BV.

## Introduction

Peer victimization at school is common among young adolescents in low, middle, and high-income countries around the globe ^1^ and is associated with a variety of negative social, academic, and mental health outcomes ^2^ such as depression, anxiety, self-harm, and attempted suicide. ^3 - 6^

The literature shows that prevalence rates of peer victimization vary widely across studies, depending on the definitions and measures adopted. A study examining victimization by bullying at school in 48 countries (predominantly low and middle-income countries) (n = 134,229; 12-15 years) showed that prevalence rates varied by country. In the region of the Americas, the overall prevalence rates of bullying victimization (BV) in the past 30 days varied from 19.1% in Costa Rica and Uruguay to 47.2% in Peru. ^7^ In Brazil, the 2009 National Adolescent School-Based Health Survey investigated BV in the past 30 days in a representative sample of 60,973 ninth grade students from 26 State capitals and the Federal District of Brasília (response rate: 88.7%). BV at school was reported by 5.4% of students, with a higher prevalence rate among boys (6.0%) compared to girls (4.8%). ^8^ Subsequently, the 2012 National Adolescent School-Based Health Survey investigated BV in the past 30 days in another representative sample of 109,104 ninth grade students from Brazil’s 26 State capitals and the Federal District of Brasília. Using the same measure, this survey found a higher prevalence of BV at school among boys (7.9%) compared to girls (6.5%), and an overall rate of 7.2%. ^9^ A meta-analytic review of sex differences in forms of aggression showed that sex differences were highest for physical aggression, were lower, but still in the male direction for verbal aggression, and were absent or in the female direction for indirect aggression. ^10^ Another meta-analytic review of overt and relational peer victimization found that boys were slightly more likely to experience overt victimization (physical, verbal), but there was no sex difference regarding relational victimization. ^11^ Developmentally, peer bullying is evident as early as preschool, increases throughout elementary school, peaks in middle school, and declines in high school. ^12 , 13^

Regarding the concept of peer victimization, this can be used as an umbrella term that includes both exposure to peer aggression (PA) and BV. Researchers generally agree that bullying is a subset of peer victimization, traditionally defined as a type of aggressive behavior by one or more individuals with intent to cause harm (injury or discomfort) to another individual (victim), and is characterized by intentionality (the perpetrator intends to harm the target person), frequency (repeated aggressive behavior as a proxy for greater harmfulness), and power imbalance between perpetrator and victim (e.g., differences in physical strength, self-confidence, popularity/status in the peer group), making it feel difficult for the victims to defend themselves. ^4^ However, studies have found that definitions of bullying developed by researchers may differ from those of young people. According to Guerin and Hennessy, ^5^ students (n = 166, 10-13 years) do not agree with researchers in respect of the importance of repetition and intention in defining bullying. Students focus more on the effect of bullying incidents on the victim, and the victim’s interpretation of the incident, than on the intention of the bully. Vaillancourt et al. ^6^ collected self-reports from 1,767 students (8-18 years) to evaluate whether the themes that emerged from the students’ definitions of bullying were consistent with theoretical and methodological operationalizations within the research literature. Whereas researchers typically emphasize intentionality, repetition, and power imbalance in their definitions, students tended to focus primarily on negative actions and rarely mentioned these three definitional criteria. In fact, the victim’s perception is the most important factor: how they assess the situation, how they react, and how they feel. ^14^

Studies of BV frequently present significant inconsistencies with respect to the measurement strategies applied. Some researchers ask the study participants if they have been repeatedly bullied, without first giving them a definition of bullying, making it difficult to understand what positive responses mean. ^15 , 16^ Others investigate BV after presenting a pre-established definition of bullying to the study participants to avoid false positive responses based on a variety of individual subjective interpretations. Students who are exposed to a previous definition of bullying report a lower rate of victimization compared to those who report bullying without receiving a prior definition of the term. ^6^

When studying general victimization among school peers, one common research strategy is to present a list of victimization-related behaviors and ask how often the youth has experienced them during a specific time, without first providing a working definition of BV. This strategy allows identification of exposure to different PA behaviors irrespective of how the adolescents are interpreting them. Youth may interpret exposure to PA as harmless play among peers or they may interpret the events as aggressive and hurtful. Because some individuals feel that the aggression suffered did not cause them harm, not everyone who suffers PA considers themselves to be victims of bullying. ^17^ Therefore, it is important to evaluate whether exposure to PA and BV are two different constructs among Brazilian adolescents, as reported by Hellström et al. ^18^ in Sweden, and Söderberg and Björkqvist ^19^ in Finland.

According to different authors, harm from the victim’s perspective is likely to be an essential component of peer victimization measures. ^14 , 20 , 21^ The measure of BV used in this study required the adolescents to feel harmed by their peer victimization experiences. However, our measure may be criticized for not requiring the presence of power imbalance, as power imbalance has been used as a criterion to separate bullying from general proactive aggression. ^20^ Nonetheless, it has been found to be difficult to operationalize and capture in assessments among children. ^17^

## Objectives

Regarding four age-sex groups (11-to-12-year-old boys, 13-to-15-year-old boys, 11-to-12-year-old girls, 13-to-15-year-old girls), the objectives of this study were: (1) to examine whether exposure to PA events (reported by adolescents without previously receiving a definition of bullying) and BV (reported after receiving a definition of bullying) may be considered distinct experiences; (2) to evaluate the extent to which adolescents exposed to PA at school consider themselves to be victims of bullying; and (3) to investigate differences among these groups in terms of the effect on BV of the number of PA events experienced.

## Methods

### Study design and sampling

This is a cross-sectional study nested in a longitudinal study (Itaboraí Youth Study) that investigated a probabilistic community-based sample of 1,409 6-to-15-year-olds at baseline (response rate = 87.8%). The study was conducted in Itaboraí, a low-income medium-size city in the state of Rio de Janeiro, southeast Brazil (218,008 inhabitants, 98% urban). ^22^ Itaboraí city is one of the poorest municipalities of the eastern portion of the metropolitan region of Rio de Janeiro state, with 71,007 inhabitants living in extreme poverty according to the last census (2010). ^23^

The Itaboraí Youth Study used a three-stage sampling procedure that first involved a random sample of census units (107/420) using the probability proportional to size method, second a random sample of eligible households (15 in each selected census unit) and third, a target child randomly selected among all eligible children in each participant household. The eligibility criteria were boys and girls aged 6-15 years residing with his/her biological, step, or adoptive mother. Exclusion criteria were intellectual disabilities (child not able to play with other children or go to a mainstream school or class) and the mother being younger than 18 years. More detailed information on the Itaboraí Youth Study methods can be found elsewhere. ^24^

The baseline sample (n = 1,409) included 720 adolescents (11-15 years), 94.4% of whom were individually interviewed (n = 680). The current paper analyses data reported by adolescents who had been attending school in the previous six months (n = 669, 51.7% girls).

### Procedures and measures

During the period from February to December, 2014, trained lay interviewers individually administered a questionnaire to adolescents (n = 680) at home under confidential conditions. The measures adopted for exposure to PA at school and for BV are described below.

#### Exposure to PA at school

A 15-item scale previously used in a Norwegian study with schoolchildren ^25^ investigated three types of PA events: physical aggression (4 items: kicking, threatening, tripping him/her up, hitting), verbal harassment (5 items: name calling, teasing, teasing about family, teasing because he/she was different, hurting feelings) and social manipulation (6 items: ganging up on him/her, making him/her hurt other people, getting him/her into trouble, making him/her do something he/she didn’t want to, threatening to tell on him/her, lying about him/her). Possible answers for all items were: “not at all” (0), “once” (1), “more than once” (2). This 15-item scale included selected and modified items from Arora’s “My Life in School” checklist. ^26^ Any PA was defined as at least one event occurring more than once in the past six months, while the number of PA events experienced by the adolescents revealed the total number of events that occurred more than once in the past six months (the 15-item scale total score ranged from 0 to 15 after responses had been dichotomized into “more than once” vs. “not at all/once”).

#### BV

The current study is focused on victims of bullying at school without discriminating victims only from bullies/victims, and without including cyberbullying. After investigating the occurrence of PA events, the interviewer informed the adolescent of the definition of bullying adopted (“when one or more school peers are repeatedly doing bad things to you such as name-calling, threatening, hitting, spreading rumors about you, excluding you from the group, or teasing you to hurt your feelings”), and then asked one question about BV: “How often have you been bullied in the past six months?” Answers were coded as “not at all” (0), “less than once a week” (1), “more than once a week” (2) or “most days” (3). A frequency of more than once a week or most days in the past six months identified repeated exposure. The general question asked to investigate BV was not restricted to the 15 PA events examined but could be related to any type of peer victimization experienced by the respondents in the past six months.

## Statistical analysis

In this paper, absolute numbers of subjects are unweighted (refer to the sample), while percentages are weighted (refer to the city population). Weighting was not used for the logistic regression analysis. Regarding all variables of interest for the current study, no missing data were registered during the interviews with the study participants.

Exposure to PA and BV were combined to generate four mutually exclusive categories (PA only, BV only, both, and none) and chi-square tests were used to verify differences in the rates of these categories between four age-sex groups (11-to-12-year-old boys, 13-to-15-year-old boys, 11-to-12-year-old girls, and 13-to-15-year-old girls). In our study, questions about exposure to PA and BV were asked to in-school adolescents aged 11-15 years. The decision of grouping 11-to-12-year-olds and 13-to-15-year-olds was based on the World Health Organization’s definition of age sub-groups of adolescents: early adolescence (10-12 years), mid adolescence (13-15 years) and late adolescence (16-19 years). ^27^

Multiple logistic regression analysis was applied to examine the effect of the number of PA events experienced on BV (study outcome), and whether this effect differed according to age and sex. Three two-way interactions (PA*age, PA*sex, age*sex) and one three-way interaction (PA*age*sex) were investigated in the analysis. When evaluating interactions, we estimated associations between PA and BV within the four age-sex groups. Statistical significance was determined by p < 0.05 except when Bonferroni correction was necessary. SPSS 20 was used for all analyses.

## Ethical considerations

All procedures performed in this study (interviews), which involved human participants, were in accordance with the ethical standards of the Brazilian National Committee for Ethics in Research (process 25000.182992/2011-76), the Research Ethics Committee of the Universidade Federal de São Paulo (process 0324/11), and the 1964 Helsinki declaration and its later amendments or comparable ethical standards. Written informed consent was obtained from mothers authorizing participation of their son/daughter and written informed assent was obtained from all participating adolescents.

## Availability of data and material

Our study has associated data in a data repository in Norway (Norwegian Centre for Research Data - https://nsd.no/nsd/english/index.html). The data supporting the findings of the article are not currently available to the public since the study data are currently restricted to the research team responsible for the study, invited research colleagues, and post-graduate students.

## Results

The current study involved a representative sample of in-school adolescents living in Itaboraí city (n = 669, 11-15 years, 51.7% girls). The mean age ± SD was similar among boys (12.9±0.1 years) and girls (13.1±0.1 years). In the past six months, 21.9% of adolescents reported exposure to one or more PA events at school, and 5.5% considered themselves victims of bullying.

### Are exposure to PA and BV identical constructs?

The current study found that 17.5% of adolescents reported only exposure to PA, 1.1% reported only BV, 4.4% reported both, and 77.1% reported neither. The four age-sex groups did not differ significantly in PA/BV distribution as shown by the overall chi-square test (p = 0.07) ( Table 1 ). For all groups, the rate of exposure to PA only significantly (Bonferroni-corrected) differed from the rate of BV only, and for all groups (except younger girls) the rate of exposure to PA only significantly differed from the rate of overlap between PA and BV (Bonferroni correction: p < 0.05/8 = 0.006) ( Table 1 ).

**Table 1:** Rates of four mutually exclusive categories of exposure to PA* and BV † combined according to four age-sex groups ‡

Four age-sex groups	Four mutually exclusive categories								PA only vs. BV only	PA only vs. Both
	PA only		BV only		Both		Neither			
	N (%)	(95%CI)	N (%)	(95%CI)	N (%)	(95%CI)	N (%)	(95%CI)	p ^§^	p ^§^
Younger boys (11-12 years, N = 133)	29 (23.1)	(15.4-33.0)	3 (1.5)	(0.5-5.0)	8 (6.8)	(2.8-15.4)	93 (68.7)	(59.5-76.6)	< 0.0005	0.0006
Older boys (13-15 years, N = 194)	39 (24.6)	(17.4-33.4)	2 (0.7)	(0.2-3.3)	6 (2.6)	(1.1-5.9)	147 (72.1)	(62.9-79.7)	< 0.0005	< 0.0005
Younger girls (11-12 years, N = 125)	14 (10.6)	(6.1-18.0)	2 (1.2)	(0.3-4.8)	9 (9.2)	(4.8-16.9)	100 (79.0)	(68.5-86.6)	0.0026	0.297
Older girls (13-15 years, N = 217)	32 (11.9)	(7.6-18.1)	3 (1.1)	(0.3-3.6)	4 (1.8)	(0.6-5.5)	178 (85.2)	(78.1-90.2)	< 0.0005	< 0.0005

Absolute numbers of subjects are unweighted (refer to the sample), and percentages are weighted (refer to the city population).

95%CI = 95% confidence interval; BV = bullying victimization; PA = peer aggression.

* At least one event occurred more than once in the past six months.

^†^ More than once a week or most days in the past six months.

^‡^ The overall chi-square test (p = 0.07) did not identify differences between the four age-sex groups in the distribution of the four mutually exclusive categories.

^§^ Chi-square tests with Bonferroni correction: p < 0.05/8 = 0.006.

When considering the adolescents victimized by peers (those who reported exposure to any PA and/or BV), the current study found that 76.2% reported exposure to PA only, 4.7% reported BV only, and 19.1% reported both. The four age-sex groups did not differ significantly in the PA/BV distribution (p = 0.17).

### Adolescents exposed to PA who considered themselves victims of bullying

Among adolescents who reported one or more PA events more than once in the last six months, 20.0% considered themselves to be victims of bullying more than once a week or most days. This rate was 22.7% for younger boys, 9.7% for older boys, 46.5% for younger girls, and 13.2% for older girls.

### Association between PA and BV: the influence of sex and age

We used multiple logistic regression analysis to test for differences between the four age-sex groups in terms of the effect on BV of the number of PA events experienced. A multiple logistic regression model was run with BV as the study outcome and three blocks of independent variables: Block 1 included three individual variables (total number of PA events experienced, age group [11-12 years vs. 13-15 years], sex [girls vs. boys]); Block 2 included all possible two-way interactions (PA*age, PA*sex, age*sex); and Block 3 included one three-way interaction (PA*age*sex). The analyses using this model revealed that this three-way interaction was significant (p = 0.006). Probing analyses conducted in the four age-sex groups helped us interpret this three-way interaction, showing a significant effect of PA on BV for all groups except for older girls ( Table 2 ). Probing analysis 1 shows that for each unit increase in PA reported by younger boys, the odds of considering themselves a victim of bullying increase by a factor of 1.47 (p = 0.001). Probing analysis 2 shows that for each unit increase in PA reported by older boys, the odds of considering themselves a victim of bullying increase by a factor of 2.01 (p < 0.001). Probing analysis 3 shows that for each unit increase in PA reported by younger girls, the odds of considering themselves a victim of bullying increase by a factor of 2.43 (p < 0.001). Probing analysis 4 shows that for the older girls there is no effect of PA on BV (p = 0.095). When looking at the odds ratios from probing analyses 1 to 4 ( Table 2 ), it can be observed that the effect of PA on BV is greater for older boys than younger boys, and greater for younger girls than older girls (effects in opposite directions). It is interesting to note that there is quite a large effect difference in girls (younger > older) and a smaller difference in the opposite direction for boys (older > younger) ( Table 2 ).

**Table 2:** Secondary logistic regression analyses probing the significant three-way interaction (PA*age*sex) in the four age-sex groups of adolescents

Four age-sex groups	N	Probing analyses	Association between number of PA events* and BV ^†^	
			OR (95%CI)	p
Younger boys (11-12 years)	133	1	1.47 (1.17-1.84)	0.001
Older boys (13-15 years)	194	2	2.01 (1.41-2.86)	< 0.001
Younger girls (11-12 years)	125	3	2.43 (1.66-3.55)	< 0.001
Older girls (13-15 years)	217	4	1.33 (0.95-1.85)	0.095

Absolute numbers of subjects are unweighted (refer to the sample).

95%CI = 95% confidence interval; BV = bullying victimization; OR = odds ratio; PA = peer aggression.

* Number of PA events occurred more than once in the past six months.

^†^ BV in the past six months (more than once a week/most days vs. less than once a week/not at all).

## Discussion

### Are exposure to PA and BV identical constructs?

Our study showed that exposure to PA and BV partially overlap and are not identical constructs among adolescents. Overall, 17.5% of adolescents reported exposure to PA only, 1.1% reported BV only, 4.4% reported both, and 77.1% reported none. The general question asked to investigate BV was not restricted to the 15 PA events examined, but could be related to any type of peer victimization experienced by the respondents. This explains why 1.1% of adolescents did not report any of the 15 PA events but nevertheless considered themselves victims of bullying. As expected, reporting BV only was rare compared to reporting exposure to PA only in the four age-sex groups. Our results are in accordance with findings reported by Söderberg and Björkqvist ^19^ who empirically explored the differences between BV and victimization by PA among 3,447 Finnish 7th and 9th grade students. BV was measured by asking students whether and in what setting (e.g., school, home, neighborhood, over the phone, online) they had been bullied within the last six months, without investigating its frequency or first providing a formal definition of bullying and, therefore, allowing the respondents to answer according to their understanding of the term. PA was defined as suffering at least one form of aggression (physical, verbal, indirect) often or very often in the last six months. The authors found that 13.2% of students reported exposure to PA only, 4.1% reported BV only, 6.4% reported both, and 76.3% reported none. The study conducted by Söderberg and Björkqvist ^19^ and the current study both noted a low rate of adolescents reporting BV only, reinforcing the idea that BV without exposure to PA may be related to exposure to peer behaviors not included in the measure of PA adopted.

When considering the adolescents victimized by peers (those who reported exposure to any PA and/or BV), the current study found that most peer-victimized adolescents (76.2%) reported exposure to PA only, a much lower rate (4.7%) reported BV only, and 19.1% reported both. The fact that a measure of exposure to PA and a measure of BV captured partly different adolescents was also noted by Hellström et al. ^18^ They conducted a study involving 1,760 students (13-15 years, grades 7-9) to examine concordance and discordance between a measure of BV and a measure of exposure to PA with respect to the number of students identified as victims. Regarding PA, students were asked “Has another student/s in school done any of the following to you during the last couple of months?”, followed by a list of five types of PA including direct and indirect forms of aggressive behavior. Frequently victimized students were those exposed to one or more PA behaviors occurring at least 2-3 times/month in the past couple of months. Regarding BV, the definition of bullying used in the Health Behavior in School-Aged Children study was presented to participants before asking the questions about the frequency of traditional bullying and cyberbullying. Traditional bullying occurring frequently (at least 2-3 times/month in the past couple of months), and cyberbullying occurring at least once or twice in the past couple of months were considered positive responses, which were combined into one single measure of bullying. The authors found that from the total number of peer victimized students, 43.6% reported repeated PA only, 13.1% reported BV only, and 43.3% reported both. This indicates that a measure of exposure to PA and a measure of BV used in isolation fail to capture many adolescents victimized by peers.

### Adolescents exposed to PA who considered themselves victims of bullying

In Finland, Söderberg and Björkqvist ^19^ found that 32% of students exposed to frequent PA (often or very often in the past six months) considered themselves to be victims of bullying. In our study, the rate of adolescents exposed to PA who considered themselves to be victims of bullying (20.0%) suggests that only a reduced proportion of individuals exposed to PA felt hurt or harmed by their peer behaviors. According to the qualitative study by Mishna et al., ^28^ aggression events can be interpreted as play among peers, with no intention of causing injury or harm. According to these authors, the way the victim feels is what determines whether the victim will consider exposure to PA events as bullying or not. Another qualitative study ^17^ found that adolescents focus on the victim’s feelings to decide whether a behavior should be defined as bullying (i.e., they include the negative experience of the victim as a criterion for defining bullying). Furthermore, in the present study, adolescents who were exposed to PA, but did not report BV may have been those who did not feel hurt or harmed by peer acts regardless of the aggressor’s intentions, those who may be hesitant to admit that they were bullied because they might associate BV with weakness, or those who suffered bullying less than once a week in the past six months (below the adopted cut-off to be classified as bullying victims). Regarding the proportion of adolescents exposed to PA who considered themselves to be victims of bullying, our study found a particularly low rate among older boys (9.7%). One possible explanation is that some study participants may be reluctant to label themselves as victims of bullying, particularly older boys who do not accept their status as a victim to avoid admitting their vulnerability, which would call into question their masculinity.

Regarding age, our study showed that among boys who reported exposure to PA, 22.7% of the younger group (vs. 9.7% of the older one) considered themselves to be victims of bullying. In fact, younger boys are often victims of older ones, since older boys are in an advantageous position compared to the younger ones, because their physical size gives them a certain advantage in aggression (imbalance of power). In addition, among girls who reported exposure to PA, 46.5% of the younger group (vs. 13.2% of the older ones) considered themselves to be victims of bullying. One hypothesis to explain this finding would be the fact that the younger ones may not have yet acquired the social and assertiveness skills to effectively deal with bullying. ^29^

### Association of PA with BV: the influence of sex and age

In this study, multiple logistic regression analysis showed that boys of any age group, and younger girls but not older girls had increased odds of considering themselves a victim of bullying for increasing levels of PA events experienced. These findings raise the question of why older girls seem not to be as affected by overall PA when it comes to being victims of bullying. Apart from the low prevalence of BV in this group, that can lead to low precision in estimating associations, older girls are probably by far the most mature group among the four age-sex group combinations. In fact, girls and older adolescents have higher levels of social and emotional competencies than boys and younger adolescents, respectively. ^30^ To address this problem, evidence-based social-emotional learning programs have been used as a method to increase resiliency and promote positive mental health in young people. ^31^ It is reasonable to suppose that older girls are more resilient to the deleterious consequences of interpersonal harm. According to Hinduja and Patchin, ^32^ resilient young people who are bullied are less likely to suffer a significant impact on their ability to learn and feel safe at school and are less likely to classify their peer victimization experiences as bullying.

### Study strengths and limitations

The study strengths encompass the rigorous study methods used; the high participation rate among eligible individuals; the use of interviews to collect data, given that a significant proportion of participants could have had difficulty reading a self-administered questionnaire; and the examination of the distinction between exposure to PA and being victimized by bullying in four age-sex groups of adolescents, based on the fact that in our study BV required the self-perception of harmful exposure, while PA did not. Since the presence of power imbalance was not formally included in the bullying measure adopted, this may be recognized as a study limitation. However, the absence of a power imbalance in the bullying measure adopted could also be viewed as a strength that allows for detection of potentially harmful aggression between individuals of relatively equal power. Other potential study limitations involve the definition of bullying adopted (“when one or more school peers are repeatedly doing bad things to you such as name-calling, threatening, hitting, spreading rumors about you, excluding you from the group, or teasing you to hurt your feelings”) which could have included more examples of PA; and the specific influences of subtypes of PA (physical aggression, verbal harassment, social manipulation) on BV, which were not investigated. In addition, skin color and other types of childhood trauma were not examined in the current study.

## Conclusion

Exposure to PA and BV at school partially overlap and should be considered as distinct experiences among adolescents, not only overall, but also among specific age-sex groups. Among those exposed to PA, the proportion of adolescents who considered themselves to be victims of bullying varies according to different combinations of sex and age, being particularly low among older boys, probably due to greater reluctance to admit a victim role. Regarding the effect on BV of the number of PA events experienced, multiple logistic regression analysis showed that boys of any age group, and younger girls but not older girls had increased odds of considering themselves a victim of bullying for increasing levels of PA events experienced (older girls appear to be less affected by PA probably due to higher social/emotional competencies to deal with interpersonal conflicts).

### Implications for practice

Health professionals and educators should bear in mind that the proportion of adolescents exposed to PA who consider themselves to be victims of bullying varies according to different combinations of sex and age. Exposure to PA and BV can be considered distinct experiences in different age-sex groups since BV involves feeling harmed by the peer behaviors experienced, while exposure to PA may be interpreted as play among peers, with no intention of causing injury or harm. When working with adolescents, it is important to identify victims of harmful PA to investigate possible deleterious effects on their school performance and mental health.

### Future research directions

Future studies should consider the possibility of improving measurement of harmful PA experiences by presenting a list of victimization-related behaviors to respondents and asking them not only about the frequency of events, but also about the extent to which they have been harmed by each peer act, as suggested by Volk et al. ^20^ In addition, investigators should collect longitudinal data and use quantitative and qualitative methods combined to increase knowledge about BV among Brazilian adolescents. In Brazil, information is also needed about the potential association between low-frequency and high-frequency peer victimization and mental health problems. Since exposure to PA in general and BV are two different constructs, it is important to further explore the possible differences between these two variables in their level of impact on the adolescents’ internalizing and externalizing problems.
